# Development of a risk score for earlier diagnosis of chronic kidney disease in children

**DOI:** 10.1371/journal.pone.0215100

**Published:** 2019-04-19

**Authors:** Paulo Cesar Koch Nogueira, Tulio Konstantyner, Maria Fernanda Camargo de Carvalho, Cristine Campos de Xavier Pinto, Isabel de Pádua Paz, Vera Maria Santoro Belangero, Marcelo de Sousa Tavares, Clotilde Druck Garcia, Oreste Angelo Ferra Neto, Káthia Liliane da Cunha Ribeiro Zuntini, Marina da Rocha Lordelo, Samira Shizuko Parreao Oi, Renata Trindade Damasceno, Ricardo Sesso

**Affiliations:** 1 Department of Pediatrics, Federal University of Sao Paulo, Sao Paulo, Brazil; 2 Hospital Samaritano de Sao Paulo, Sao Paulo, Brazil; 3 Escola de Economia de Sao Paulo, Fundação Getúlio Vargas, Sao Paulo, Brazil; 4 Department of Pediatrics, State University of Campinas, Campinas, Brazil; 5 Department of Pediatrics, Federal University of Minas Gerais, Belo Horizonte, Brazil; 6 Department of Pediatrics, Federal University of Health Sciences of Porto Alegre, Porto Alegre, Brazil; 7 Hospital Universitário Maria Aparecida Pedrossian, Campo Grande, Brazil; 8 Hospital Infantil Albert Sabin de Fortaleza, Fortaleza, Brazil; 9 Hospital Martagão Gesteira, Salvador, Brazil; 10 Federal University of Maranhao, Sao Luis, Brazil; 11 Santa Casa do Pará, Belém, Brazil; 12 Nephrology Division, Federal University of Sao Paulo, Sao Paulo, Brazil; Istituto Di Ricerche Farmacologiche Mario Negri, ITALY

## Abstract

**Objective:**

To develop a clinical score for the early identification of chronic kidney disease (CKD) in children and adolescents. The early diagnosis of CKD in childhood allows the adoption of measures to slow the progression of the disease, thereby reducing morbidity and mortality. Nevertheless, the diagnosis is often made too late for proper patient management.

**Study design:**

We preformed a case-control study of a multicenter Brazilian sample of 752 pediatric patients; the study cases (n = 376) were CKD patients with a median estimated GFR of 37 (IQR = 22 to 57) ml/min/1.73 m^2^. The control group (n = 376) comprised age-, gender- and center-matched children who were followed for nonrenal diseases. Potential risk factors were investigated through a standard questionnaire that included symptoms, medical history, and a clinical examination. Two multivariable models (A and B) were fitted to assess predictors of the diagnosis of CKD.

**Results:**

In model A, 9 variables were associated with CKD diagnosis: antenatal ultrasound with urinary malformation, recurrent urinary tract infection, polyuria, abnormal urine stream, nocturia, growth curve flattening, history of hypertension, foamy urine and edema (c-statistic = 0.938). Model B had the same variables as model A, except for the addition of the history of admission during the neonatal period and the exclusion of antenatal ultrasound variables (c-statistic = 0.927).

**Conclusions:**

The present scores may serve as a warning sign for CKD diagnosis in children among professionals working in the primary care setting where the symptoms associated with a risk of CKD may be overlooked.

## Introduction

Chronic kidney disease (CKD) in children is a major disorder that leads to health system overload and represents a challenge, mainly in developing countries [1–3]. This disease usually progresses to the loss of kidney functions, leading to the need for renal replacement therapy (RRT), which has a huge impact on patients’ health conditions and family dynamics [4,5]. Particularly in children, CKD is associated with malnutrition, which stunts and delays development, causing a significant impairment in the quality of life and reduction in life expectancy [6,7].

The early diagnosis of CKD is an aim pursued throughout the world and some countries have adopted a screening strategy for the early diagnosis of CKD recommending the entire population of children to undergo a urine test at an early age [8,9]. The cost-effectiveness of this approach has not yet been proven [10].

A previous study revealed that the primary diagnosis of CKD in Brazil is late and is often confirmed when patients are close to needing chronic dialysis therapy [11]. Late diagnosis of CKD entails three potential consequences to patients: many of them might die from preventable complications of CKD without having had a definitive diagnosis; many of them did not underwent measures in the early stages of the disease to reduce the rate of CKD progression; and many of them did not have adequate preparation time and organization of the families for RRT initiation, as well as for the health facilities, which can ultimately lead to higher morbidity and mortality [7,12,13].

To address the current dilemma characterized by late and missed diagnoses of CKD in pediatrics, alongside the uncertainty about the cost-effectiveness of screening strategies based on laboratorial exams, we hypothesized that a clinical score could be a useful strategy to better select the patients more likely to benefit from laboratory/image tests to confirm the CKD diagnosis. Therefore, the aim of this study was to develop a clinical score for the early identification of children and adolescents with CKD.

## Materials and methods

### Design, study population and procedures

We performed a case-control study composed of a sample of pediatric patients under 19 years from all regions in Brazil and conducted from October 2015 to February 2017. The study was performed in eight pediatric centers.

We estimated a minimum sample of 350 cases and 350 controls. This number of subjects was based on the planned statistical analysis (multiple logistic regression), which was designed to allow the inclusion of up to 35 variables in the multivariable statistical model. The proportion of cases per region was based on the distribution of the total number of cases of End-stage renal disease (ESRD) in patients up to 19 years of age in each Brazilian region [11].

The inclusion criteria of cases were: to have a diagnosis of CKD based on the estimated glomerular filtration rate (eGFR) lower than 90 ml/min/1.73 m^2^ according to the Schwartz equation [14] and having been followed at the pediatric nephrology service for a minimum of 3 months. The exclusion criteria were: refusal to participate, guardians unable to provide data for the research, CKD caused by unpreventable illness (e.g., trauma, nephrectomy due to cancer treatment or rapidly progressive glomerulonephritis), and acute kidney injury.

The subjects in the control group were age (±2 years) and gender-matched to the cases. The enrolled controls were patients treated for nonnephrological diseases at the same center where the cases were recruited. The exclusion criteria for the control children were: a) any urine abnormality detected by a dipstick test; b) malnutrition, defined by a value of Z-score less than 2 SD in body mass index-for-age or length/height-for-age, and c) children with any chronic disease.

### Measures

Data were collected at the referral center from both patient record reviews and personal interviews with the guardians. A structured questionnaire was used. The questions included in the questionnaire were created based on consensus meetings among the coauthors. The questionnaire contained 100 questions about socioeconomic and demographic characteristics (20 questions), gestational history (14 questions), neonatal history (13 questions), personal background (31 questions) and family history (22 questions). Among the 100 questions, 57 were considered as potential identifiers for the diagnosis of CKD. In these 57 questions, the possible answers were either quantitative or categorical, comprising three categories: ‘yes’, ‘no’ and ‘I do not know’ (S1 Table). For the cases, the questions referred to the period prior to the CKD diagnosis. To ensure uniform procedures, a guidebook was created giving the interviewers a set of norms and definitions to fill out the questionnaire.

The study subjects also underwent measurements of weight, height and blood pressure. Z-scores were used to quantify nutritional disorders (stunting, wasting, and overweight). Nutritional status was defined in conformity with the Multicentre Growth Reference Study (MGRS) standards for age and sex, as recommended by the World Health Organization in 2006 [15].

### Statistical analysis and screening index generation

Univariate descriptive statistics were performed for continuous and categorical variables. To fit the model of the score, we first selected the most relevant potential risk factors among the 57 questions of the questionnaire. This selection was based on the initial descriptive analysis of the answers and clinical plausibility, as judged by consensus among the authors. Thirty variables/questions were selected at this stage. Then, we performed univariate logistic regression analyses that contained a dummy variable that indicated if the individual was a case or control as the outcome and the 30 potential predictive factors for CKD diagnosis. We subsequently constructed a multivariable logistic regression model by initially preselecting all variables that were associated with the outcome with a p value < 0.10 in the univariate analysis. Next, we fitted two multivariable models by adopting the ‘change-in-estimate’ as the criterion to select variables for the models, as follows: a) model A–Based on the statistical significance in the univariate analysis and on background clinical knowledge, we first selected the two variables with higher statistical and clinical significance and forced them into the model; then, we manually tested each further preselected variable, choosing those that when entered resulted in at least a 10% change in the estimated odds ratio of the outcome to remain in the model. After this, we inserted all previously selected variables and manually removed one-by-one the variables that did not exhibit a statistically significant association with CKD diagnosis (manual backward stepwise selection); b) model B–Based on the fact that antenatal ultrasound could not be regularly available in some settings we excluded this variable from the analysis and repeated the procedures described for the selection of variables in model A [16].

We used the following strategy to construct the predictive score. Each coefficient of our final logistic model was divided by the sum of the coefficients, so it represents the proportion of the correlation of that specific explanatory variable in relation to the others. The sum of these normalized coefficients is equal to one. Then, we estimated the fitted values for each individual using these normalized coefficients, and these modified fitted values represent the values of the model. To simplify the interpretation of the score, we normalized it to be between zero and one. Since all the explanatory variables used in the construction of the model increase the probability of being a case, the higher the value (closest to one), the higher the risk of being a case [16].

Using the same data set that was used to build the models, the internal validation of the models was performed using reanalysis techniques (bootstrap procedure). We created 5,000 resamples, and estimated the model based on the variables selected in our stepwise procedure for each one of the 5,000 samples. This procedure generates an empirical distribution of the moments of the model (mean, variance and percentiles) and if our model has a good internal validity, we expect the moments of our original model to be close to the moments of this empirical distribution. We calculated the mean bias and the coverage to analyze how close the moments of the model were to the moments of the empirical distribution. Using a hypothesis test of size 5%, the coverage probability is expected to be 95%.

The Stata statistical package version 15.1 (College Station, TX: StataCorp LP) was used to analyze the data.

### Ethical approval

This research adhered to the ethical principles of Declaration of Helsinki. Guardians of the children provided signed informed consent for data use. The Ethics and Research Committees of the Hospital Samaritano de São Paulo (n° 1.182.369) and of all the eight institutions participating in the project approved this consent.

## Results

The sample is composed of 752 children, 376 with CKD and 376 controls. The case group consisted of children with median eGFR = 37ml/min/1.73 m^2^ (IQR = 22 to 57). The causes of CKD were: congenital anomalies of the kidney and urinary tract (CAKUT) in 180 individuals (48%), unknown in 42 (11%), focal segmental glomerulosclerosis in 35 (9%), cystic kidney disease in 35 (9%), acute renal failure sequel in 11 (3%), other glomerulopathies in 10 (3%), and other diseases in 63 (17%) children. With regard to CKD stages, the patients were categorized as follows: 81 (21%) in stage 2, 150 (40%) in stage 3, 101 (27%) in stage 4 and 44 (12%) in stage 5. Median (IQR) time between the diagnosis of CKD and the application of the questionnaire was 4.1 (1.5 to 8.2) years. Table 1 shows the demographic and clinical data of the study groups.

**Table 1:** Demographic and clinical characteristics of children and adolescents with CKD and controls.

Variable	Cases (n = 376)	Controls (n = 376)	p-value
Male sex	228 (61)	223 (59)	0.644
Caucasian ethnicity	190 (51)	123 (33)	<0.001
Living in urban area	307 (82)	315 (84)	0.393
Lower social class (C-E)	300 (80)	283 (76)	0.197
Public health institution/dialysis center	369 (99)	367 (98)	0.132
Stunting	139 (37.0)	0 (0)	<0.001
Wasting	26 (6.9)	0 (0)	<0.001
Overweight	85 (22.6)	122 (32.4)	0.003
Age (yrs)	8.7 (5.0)	8.8 (4.5)	0.790
Birth weight (kg)	2.9 (0.7)	3.2 (0.6)	<0.001
Gestational age (weeks)	37.6 (2.9)	38.4 (2.4)	<0.001
Current weight (kg)	28.2 (17.0)	32.1 (17.6)	0.002
Current height (cm)	119.7 (30.7)	128.0 (27.8)	<0.001
Systolic blood pressure (mmHg)	100 (16)	99 (12)	0.108
Diastolic blood pressure (mmHg)	64 (13)	62 (10)	0.072

Values in the table are expressed as n (%) or mean (SD)

We fitted two models, as follows: In model A, 9 variables were selected: having an antenatal ultrasound with urinary malformation, recurrent urinary tract infection, polyuria, abnormal urine stream, nocturia, growth curve flattening, history of hypertension, foamy urine and facial/lower limb edema. In model B, we excluded the antenatal ultrasound variable from the testing procedures; 9 other variables showed a significant contribution to the CKD diagnosis, including the same variables from model A, with the addition of the history of admission during neonatal period variable. After fitting the final regression models we used the “collin” command in Stata to check for multicollinearity. This procedure calculates the variance inflation factor (VIF) and variables with VIF values lower than 3 can be accepted in the model. The higher VIF value obtained was 1.6, which indicates that multicollinearity was not present in both models.

Table 2 shows the regression coefficients of the factors that remained as significant predictors of the diagnosis of CKD in both multivariable models. Having a history of urinary tract malformation on antenatal ultrasound and history of recurrent urinary tract infection presented the greatest coefficient in models A and B, respectively.

**Table 2:** Factors associated with CKD diagnosis according to multivariable models results.

Model A	Coefficient	SE	p
Urinary tract malformation on antenatal US	4.434	0.774	<0.001
Recurrent urinary tract infection	3.055	0.370	<0.001
Growth curve flattening	2.709	0.523	<0.001
Polyuria	1.810	0.482	<0.001
Nocturia	1.394	0.355	<0.001
Abnormal urine stream	1.847	0.649	0.004
History of hypertension	1.923	0.356	<0.001
Foamy urine	1.577	0.427	<0.001
Edema	1.262	0.350	<0.001
Constant	-2.556	0.201	<0.001
Model B	Coefficient	SE	p
Recurrent urinary tract infection	2.922	0.357	<0.001
Growth curve flattening	2.640	0.506	<0.001
Polyuria	1.904	0.448	<0.001
Nocturia	1.143	0.339	0.001
Abnormal urine stream	2.012	0.582	0.001
History of hypertension	1.747	0.348	<0.001
Foamy urine	1.534	0.416	<0.001
Edema	1.046	0.341	0.002
Neonatal admission	0.913	0.267	0.001
Constant	-2.453	0.194	<0.001

US = ultrasound, SE = Standard Error.

Model A: urinary tract malformation on antenatal ultrasound, recurrent urinary tract infection and growth curve flattening were named variables A1; polyuria, nocturia, abnormal urine stream, history of hypertension, foamy urine and edema were named variables A2.

Model B: recurrent urinary tract infection and growth curve flattening were named variables B1; polyuria, nocturia, abnormal urine stream, history of hypertension, foamy urine, edema and neonatal admission were named variables B2

For model A, the sensitivity was estimated to be 86%, specificity 91%, and overall correct classification of cases 88%. For model B, the sensitivity was 84%, specificity 89%, and overall correct classification of cases 86%.

**Fig 1:**
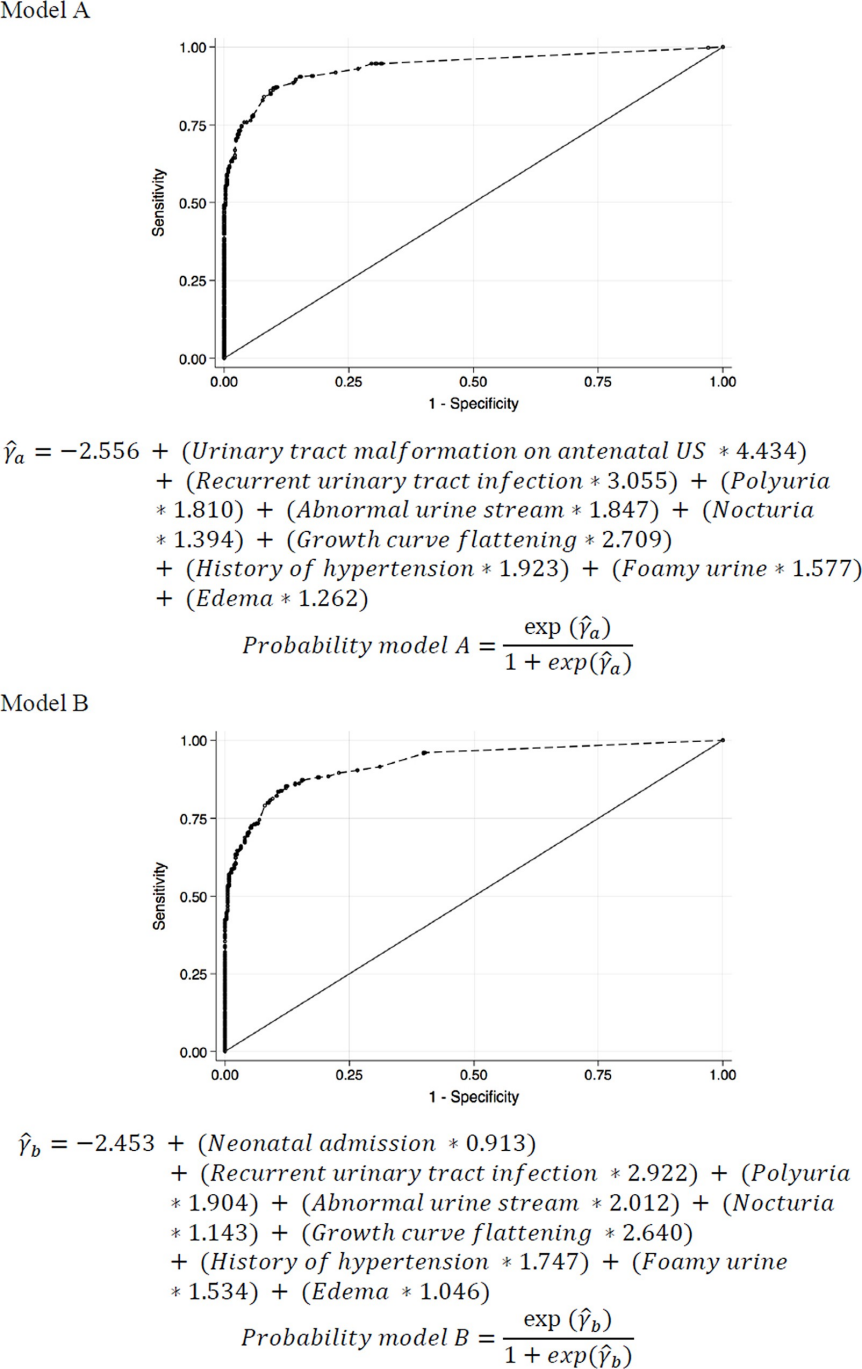
Receiver operating characteristic (ROC) curves for two multivariable models for CKD in children and adolescents (A and B).

Fig 1 shows the receiver operating characteristic (ROC) curves for both models. Model A had an area under the curve = 0.938 (95% CI = 0.920 to 0.955) and Model B had an area under the curve = 0.927 (95% CI = 0.910 to 0.946). The equations to predict the probability of CKD derived from both models are shown in Fig 1. Note that for each parameter, the respective coefficient should be multiplied by 1 if present or by 0 if absent.

Table 3 shows the results of the bootstrap analyses to verify the internal validity of the models. There was a relatively low internal validity for model A since the biases of the moments are not too small and the coverage is far from 95%. The internal validity of the model B is better, the biases are smaller and the coverage is approximately 95%. These results indicate that model B is more stable than A.

**Table 3:** Results of the internal validation (bootstrapping) of models to identify children and adolescents at risk of CKD diagnosis.

	Model A		Model B	
	Bias	Coverage	Bias	Coverage
Average	0.09	0.59	0.04	0.92
SD	0.07	0.43	0.01	0.98
P 25%	0.03	0.93	0.02	0.93
P 50%	0.07	0.77	0.03	0.93
P 75%	0.14	0.42	0.05	0.93

## Discussion

The main contribution of this study was the establishment of clinical signs or symptoms that may be useful to identify children at a greater risk of having a CKD diagnosis. The factors that were selected as predictors of CKD diagnosis in our score could be categorized into 4 groups: a) CAKUT indicators (antenatal ultrasound with urinary tract abnormality, history of recurrent urinary tract infection and abnormal urine stream); b) growth interruption (flat growth curve); c) loss of urinary concentration symptoms (polyuria and nocturia) and d) early neonatal diseases (neonatal admission). All the signs sorted out from the models have a plausible biological role to function as risk markers, and none of them is per se a new discovery. We postulate that the score here described allows one to anticipate the diagnosis and to quantify the risk of CKD in pediatric patients, thereby yielding the support for further detailed laboratory examination.

The magnitude of the regression coefficients in model A shows that the influence of each variable is not uniform with regard to the probability of being associated with CKD diagnosis. Accordingly, 3 variables (urinary tract malformation on antenatal ultrasound, recurrent urinary tract infection and growth curve flattening, here named variables A1), separately, were associated with a risk higher than 50% of having CKD and any combination of two of these A1 variables results in a chance greater than 90% of CKD. The other six variables (polyuria, nocturia, abnormal urine stream, history of hypertension, foamy urine and edema, here called A2 variables), are one by one, associated with a risk of CKD lower than 50% and any combination of 3 of these A2 variables results in a risk greater than 90% of CKD. A risk greater than 90% was also observed with any combination of one A1 variable with two A2 variables.

In model B, similarly, there were two variables associated with a risk greater than 50% of having CKD (recurrent urinary tract infection and growth curve flattening, named variables B1), while the other seven were associated with less than 50% of chance. Again, two B1 variables, or one B1 plus two B2 variables, or yet three B2 variables yielded scenarios associated with a CKD risk greater than 90%.

A score to earlier detect CKD in children is logically shaped by the etiologic profile of nephropathies in the region where it was generated. There are particularities in the etiologic profile of nephropathies in each region of the world, and one of the most notable cases is from Finland, where the prevalence rate of congenital nephrotic syndrome is higher [17,18]. The main etiology in the present sample was CAKUT in 48% of the cases, so symptoms that indicate this etiology would logically stand out in the score. The etiologic profile of our sample is similar to that reported in other cohorts of children with CKD in developed countries in general, which suggests that the present predictive score could be generalized to other regions and nations [19–23]. However, the number of individuals with undetermined etiology in our sample (11%) is higher than in other studies, and this is exactly one additional reason to justify the need of the present research, since a portion of the diagnoses of unknown cause could reflect a delay in suspecting a diagnosis.

An interesting finding was some signs that were not associated with the diagnosis in our sample, despite being generally considered as potential markers of CKD. Examples of these factors are oligohydramnios, polyhydramnios, malformations other than in urinary tract and the coexistence of any type of syndrome. Perhaps this fact was caused by the relative rarity of these other signs. Another notable absence was the lack of association between birth weight and the diagnosis of CKD in our models, despite the previously reported evidence in this regard. To explain this discrepancy, we presume that a longer follow-up would be necessary to disclose the effects of low birth weight on renal function. It is likely that the clinical effects of low birth weight are apparent only in the long-term during adulthood [24,25].

Another feature of our study was that the loss in urinary concentration capacity stood out as one of the factors for the early diagnosis of CKD. This result agrees with the reports by Garcia-Nieto et al, who also found that loss in renal concentrating capacity was a marker of GFR impairment in a group of children with CKD [26,27]. This finding may be due to a lack of responsiveness of the principal cells in the distal tubules to vasopressin action as suggested by Pedersen et al. [28]. These authors found that patients with CKD have reduced renal concentrating and diluting capacities compared to healthy control subjects. They attributed these findings to an abnormally decreased response in the AVP-c-AMP-AQP2 axis [28].

An additional notable sign observed in both of our models was growth retardation. Many different causes have been shown to contribute to growth retardation in children with CKD [29], but our results suggest that this symptom may occur early and should be considered as an alert sign for diagnosis of the disease.

Neonatal admission was the last sign included in our model, which was unveiled only when we removed the antenatal ultrasound from the model. The question about this event was generically formulated, denoting the admission in the neonatal period (up to the 28th day of life). We hypothesize that this finding indicates the effect of early health problems in the long-term and, to the best of our knowledge, this is the first time that this variable has shown an association with CKD diagnosis.

The predictive models generated in the current investigation exhibited a very good accuracy. These findings suggest that the models may be useful tools to earlier identify cases in general clinical pediatric settings that should undergo a more detailed laboratory assessment.

There are different ways to determine the internal validation of a model. The best known methods used to estimate the predictive perform of an estimator are bootstrap, cross-validation and in-sample validation [30]. In this research, we evaluated the performance of the models using a bootstrap technique, which, as argued by Steyerberg et al. [30], is the most efficient validation method.

Several limitations must be considered in the interpretation of the present research. First, case-control studies are subject to loss of recall, mainly in individuals with long-term CKD diagnosis. In this study the median time elapsed between the diagnosis of CKD and the interview was 4.1 years. The objectivity of the questions and the confirmation of imprecise responses checking patients’ medical chart helped to minimize this possible source of bias. Another limitation is that not all the controls underwent serum creatinine measurement. This was due to the ethical concern of not subjecting healthy children to invasive procedures solely because they agreed to participate in the research. However, all subjects in the control group had a complete clinical evaluation, a normal dipstick test, and in a subgroup of 154 controls (41%) that had recently performed serum creatinine measurements, we observed a mean eGFR of 123 ml/min/1.73 m^2^ (95% CI = 118 to 129).

On the other hand, a strength aspect of our study is that this multicenter sample of patients was representative of the whole country. Considering that Brazil is a developing country, we believe that our sample must be consonant to other children with CKD living in countries with similar socioeconomic conditions, which is the case for many other regions.

In conclusion, out of almost a hundred of the signs surveyed, a set of symptoms with easy clinical verification emerged as an indicator of risk for CKD in children. Professionals working in primary care are often overloaded and it is possible that the CKD signs/symptoms may go undetected. For this reason, the present score may serve as a warning sign for CKD diagnosis in children. We believe that this score should be seen as an educational tool, which does not diminish its potential significance, especially in situations in which the diagnosis of CKD is often overlooked. More research should be done to validate this approach in other populations.

## Supporting information

S1 Table Structured field questionnaire. (DOCX)

S1 Dataset (DTA)
