# Supplementary material for: Development of a risk score for earlier diagnosis of chronic kidney disease in children
Source: PLoS One. 2019 Apr 19;14(4):e0215100. doi: 10.1371/journal.pone.0215100 (PMC6474594; doi:10.1371/journal.pone.0215100)
Supplement: S1 Table — (DOCX) [file pone.0215100.s001.docx]

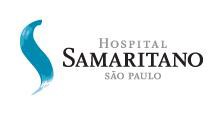

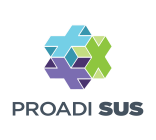


**Project “**Strategies in order to diminish regional differences in pediatric renal transplant in Brazil" - **Subproject 1** "Building a score for the early diagnosis of chronic renal disease in children"".

***Instrumentation*** Sample group: Case (1) Control (2)

*If control, Dipstick: Normal (1) Altered (2)*

Nephrology Center: ___________________________________________________________

Interviewer _____________________________ Date of the sample collection ____/____/______

# IDENTIFICATION AND SOCIOECONOMIC AND DEMOGRAPHIC CHARACTERISTICS

1. Initials: _____________________ RH: ___________________________ ID:

2. DOB: ____/____/______ 3. Gender: Male (1) Female (2) Undefinied (3)

4. Race: Caucasian (1) Black (2) Asian (3) Pardo Brazilian (4) Other (6)

5. Nationality: _________________ 6. County of residence: _______________________

7. State of residence: ___________________ 8. Zone of residence: City (1) Countryside (2)

9. Economic classification (ABEP): Scoring Class

10. Mother's age ________ years 11. Mother's education: ________ years

12. Father's age ________ years 13. Father's education: ________ years

14. Adopted Children? Yes (1) No (2) 15. Age at adoption: _______ years

16. Service center: Public (1) Private (2) 17. N.º of people at residence : ______

18. N.º of siblings who live together: 1 (1) 2 (2) 3 (3) 4 (4) >4 (5) None (6)

19. N.º of deceased children (same mother) 1 (1) 2 (2) 3 (3) 4 (4) >4 (5) None (6)

20. N.º of siblings (under 5 years): 1 (1) 2 (2) 3 (3) 4 (4) >4 (5) None (6)

**GESTATIONAL BACKGROUND**

21. Clinical complications during pregnancy? Yes (1) No (2) Don't know (3)

22. Which? ____________________________________________________________________

23. Use of medication during pregnancy? Yes (1) No (2) Don't know (3)

24. Which? ____________________________________________________________________

25. Use of Illegal drugs during pregnancy? Yes (1) No (2) Don't know (3)

26. Previous abortions? Yes (1) No (2) Don't know (3)

27. Smoking during pregnancy? Yes (1) No (2) Don't know (3)

28. Alcohol during pregnancy? Yes (1) No (2) Don't know (3)

29. Alterations in prenatal ultrasound? Yes (1) No (2) Don't know (3)

30. If yes, check with an "X" the option in the table below which corresponds to the ultrasound alteration:

| ***Alteration*** | ***Yes*** | ***No*** | ***Don't know*** |
| --- | --- | --- | --- |
| Oligohydramnios |  |  |  |
| Polyhydramnios |  |  |  |
| Urinary system structure |  |  |  |
| Any other organ/system structure alteration |  |  |  |
| If yes, which organ/system? _______________________________________________ | | | |

# PRENATAL BACKGROUND

| 31. Weight: ____ . __________ g | | Don't know (99) | | |  |
| --- | --- | --- | --- | --- | --- |
| 32. length: ______ cm | | Don't know (99) | | |  |
| 33. Gestation length: _____ weeks | | Don't know (99) | | |  |
| 34. Apgar score at 5th minute: _________ | | Don't know (99) | | |  |
| 35. Episodes at birth | ***Yes*** | | ***No*** | ***Don't know*** | |
| Hospitalization at birth |  | |  |  | |
| Neonatal antibiotic |  | |  |  | |
| Umbilical Catheter |  | |  |  | |
| Lumbosacral malformation |  | |  |  | |
| Genital malformation |  | |  |  | |
| Ear malformation |  | |  |  | |
| Extremities malformation |  | |  |  | |
| Other malformation |  | |  |  | |
| If so, which malformation(s)? _________________________________________________ | | | | | |

**PERSONAL BACKGROUND** (prior to chronic renal disease diagnosis)

1. Length of breastfeeding: __________ months Don't know (3)
2. Genetic Syndrome (any sorts): Yes (1) No (2) Don't know (3)
3. If so, which? ______________________________________________________________

| 39. Clinical characteristics | ***Yes*** | ***No*** | ***Don't know*** |
| --- | --- | --- | --- |
| Orally administered iron-resistant anemia |  |  |  |
| Eye Disease |  |  |  |
| Hearing impairment |  |  |  |
| Hospitalization on 1st day of life |  |  |  |
| Acquired bone deformity |  |  |  |
| ICU admission |  |  |  |
| Recurrent UTI |  |  |  |
| Renal lithiasis |  |  |  |
| Polyuria |  |  |  |
| Dysuria |  |  |  |
| Polydipsia (excessive thirst) |  |  |  |
| Abnormal urine stream |  |  |  |
| Nocturia |  |  |  |
| Day time urine loss |  |  |  |
| Weight gain interruption |  |  |  |
| Growth interruption |  |  |  |
| Primary monosymptomatic nocturnal enuresis |  |  |  |
| Secondary monosymptomatic nocturnal enuresis |  |  |  |
| History of hypertension |  |  |  |
| Current use of antihypertensive drugs |  |  |  |
| Red urine (bloody or dark) |  |  |  |
| Foamy urine |  |  |  |
| History of facial or lower limbs edema |  |  |  |
| Chronic intestinal constipation |  |  |  |
| Convulsion crisis |  |  |  |
| History of dialysis |  |  |  |
| History of surgery |  |  |  |
| If so, which surgery(ies) ? ____________________________________________________ | | | |

# FAMILY BACKGROUND

1. Parents age at birth of child: Mother: ________years Don't know (9)

Father: ________ years Don't know (9)

1. Consanguinity between parents Yes(1) No (2) Don't know (9)

| 42. History of CKD, Dialysis or Transplantation | *Yes* | *No* | *Don't know* |
| --- | --- | --- | --- |
| Parents |  |  |  |
| Siblings |  |  |  |
| Grand-Parents |  |  |  |
| Uncles |  |  |  |
| Cousins |  |  |  |
| If so, which etiology(ies)? ___________________________________________________  ____________________________________________________________________________ | | | |

| 43. History of Glomerulonefrite | *Yes* | *No* | *Don't know* |
| --- | --- | --- | --- |
| In parents |  |  |  |
| In siblings |  |  |  |
| In grand-parents |  |  |  |
| In uncles |  |  |  |
| In cousins |  |  |  |
| If so, which etiology(ies)? ___________________________________________________  ___________________________________________________________________________ | | | |

| 44. History of other renal pathology | *Yes* | *No* | *Don't know* |
| --- | --- | --- | --- |
| In parents |  |  |  |
| In siblings |  |  |  |
| In grand-parents |  |  |  |
| In uncles |  |  |  |
| In cousins |  |  |  |
| If so, which? _____________________________________________________________  ___________________________________________________________________________ | | | |

| 45. History of other alterations or diseases in family? | *Yes* | *No* | *Don't know* |
| --- | --- | --- | --- |
| In parents |  |  |  |
| In siblings |  |  |  |
| If so, which? _____________________________________________________________  ___________________________________________________________________________ | | | |

*Mention diabetes, cardiovascular disease (ex: hypertension, heart attack/angina, CABG, cerebral ischemia/stroke, peripheral vascular disease).

# CURRENT CLINICAL ASPECTS - physical examination

46. Current weight: ________ . ___________ grams 47. Current height: _________ cm

Blood pressure (sitting) in mmHg: 48. PAs_1_ = __ __ __ PAd_1_ = __ __ __

49. PAs_2_ = __ __ __ PAd_2_ = __ __ __ 50. PAs_3_ = __ __ __ PAd_3_ = __ _**_ __**

| 51. Malformations | *Yes* | *No* |  | *Yes* | *No* |
| --- | --- | --- | --- | --- | --- |
| Lumbosacral |  |  | Ears |  |  |
| Genital |  |  | Extremity |  |  |

1. Other malformation? Yes (1) No (2)
2. If so, which? ____________________________________________________________

**Economic Classification Criterion Brazil - 2015 (ABEP)**

This form is intended to write down the points related to the ABEP 2015 economic classification. Use it in order to simplify the final scoring assessment when using the Orientation Guide. The sum of the points and the classification must be registered in item number 9 in the Field Instrument part.

Child's RH: ___________________________ ID: **__________**

| **Items - Income power** | **Quantity existent at residence** | **Points** |
| --- | --- | --- |
| Bathroom |  |  |
| Housekeeper |  |  |
| Car |  |  |
| Computers |  |  |
| Dish-washer |  |  |
| Fridge |  |  |
| Freezer |  |  |
| Washing machine |  |  |
| DVDs |  |  |
| Microwave oven |  |  |
| Motorcycle |  |  |
| Clothes dryer |  |  |
| **Total (A)** |  |  |

| **Item - Education of the person of reference** | **Points** |
| --- | --- |
| **Total (B)** |  |

| **Items - Public Services** | **Points** |
| --- | --- |
| Tap water supply |  |
| Paved street |  |
| **Total (C)** |  |

| **Total of points (A + B + C)** |  |
| --- | --- |
| **Classification (Economic)** |  |
